# The support of early-career researchers in health professions education—an expert position statement

**DOI:** 10.3389/fmed.2025.1621194

**Published:** 2025-06-19

**Authors:** Doreen Herinek, Franziska Matthes, Mohamed Al-Eraky, Elizabeth Anderson, Julie Browne, Maria Cassar, Ingrid Darmann-Finck, Götz Fabry, Marion Huber, Fiona Kent, Mirjam Körner, Sylvia Langlois, Kristina Mikkonen, Elise Paradis, Lisa Quinn, Ara Tekian, Daniëlle Verstegen, Robyn Woodward-Kron, Michael Ewers

**Affiliations:** ^1^Institute of Health and Nursing Science, Charité – Universitätsmedizin, Berlin corporate member of Freie Universität Berlin and Humboldt-Universität zu Berlin, Berlin, Germany; ^2^Institute of Health Workforce Development, Gulf Medical University (GMU), Dubai, United Arab Emirates; ^3^Leicester Medical School, College of Life Sciences, The University of Leicester, Leicester, United Kingdom; ^4^School of Medicine, Cardiff University, Wales, United Kingdom; ^5^Faculty of Health Sciences, University of Malta, Msida, Malta; ^6^Institute for Public Health and Nursing Research, University of Bremen, Bremen, Germany; ^7^Institute for Medical Psychology and Medical Sociology, University of Freiburg, Freiburg, Germany; ^8^Center for Interprofessional Learning and Practice IPLP, School of Health Sciences, Institute of Public Health IPH, Zurich University of Applied Sciences (ZHAW), Winterthur, Switzerland; ^9^Royal College of Surgeons in Ireland, University of Medicine and Health Sciences, Dublin, Ireland; ^10^Institute for Collaborative Practice and Leadership in Healthcare, University of Applied Sciences Bern, Bern, Switzerland; ^11^Centre for Advancing Collaborative Healthcare and Education, University Health Network and University of Toronto, Toronto, ON, Canada; ^12^Research Unit of Health Sciences and Technology, University of Oulu, Oulu, Finland; ^13^NONE, San Francisco, CA, United States; ^14^College of Medicine, University of Illinois, Chicago, IL, United States; ^15^School of Health Professions Education, Faculty of Health, Medicine, and Life Sciences, Maastricht University, Maastricht, Netherlands; ^16^Department of Medical Education, Melbourne Medical School, University of Melbourne, Melbourne, VIC, Australia

**Keywords:** health professions education, early-career researchers, position statement, medical education, career pathways

## Abstract

**Introduction:**

The development of health professions education (HPE) as an academic discipline requires well-qualified educational researchers, equipped with the competence to advance the field. There is, therefore, a need to establish and support pathways in which early-career researchers (ECRs) can develop the necessary competence to pursue a career in this field.

**Approach:**

A group of 19 international experts in HPE from various professions, conducted a 2.5-day Scoping Workshop in Hannover, Germany, in November 2024. The main output of the workshop is a joint position statement on the support of ECRs in HPE, using appreciative inquiry and collaborative writing.

**Position:**

The Scoping Workshop led to a dynamic and productive exchange of ideas and experiences resulting in a common vision and five positions: (1) identify, establish, and recognize distinct career paths, (2) develop and implement a robust funding strategy, (3) create a nurturing and diverse intellectual culture, (4) connect research to practice and address real-world problems, (5) invest in leadership, advocacy, and coaching. There was strong agreement that these areas were not well developed and required urgent attention.

**Outlook:**

There is a need to foster interprofessional and interdisciplinary collaboration and provision of sustainable support structures so that ECRs can advance HPE. Only when these areas are addressed can these educational researchers contribute to the development of effective learning which prepares the healthcare workforce to meet today’s challenges. Researchers, educators, decision-makers and stakeholders in academia, education, and health and social care contexts share a responsibility for shaping the way forward.

## 1 Introduction

There is an international demand for an expanded, and well-trained health professional workforce within the setting of finite resources (1) as health professions education (HPE) makes a direct contribution to a more and more complex healthcare. There is evidence that HPE has the potential to strengthen the knowledge and skills of professionals, promote interprofessional collaboration (2, 3), and lead to better patient outcomes and safer care (4). Hence, the question arises about how to best educate the future workforce for practice challenges. High-quality health professions education research (HPER) is required to direct education and address the evolving complexities of clinical practice (5, 6). HPER is an independent and highly applied field of research that profits from the interplay of multiple areas and disciplines [(7) p. 6]. This research deals with the education and training of health professionals to enhance educational practice, policy and outcomes. Due to the call for HPE to be theory-driven (8), and evidence-based (9, 10), existing expertise and practices require review, and new generalizable knowledge needs to be generated through HPER to meet current and future challenges (10–13).

Among others, early-career researchers (ECRs) play a crucial role in advancing HPE (10). The term ECR is not uniformly defined (14). In our context, ECR refers to those beginning their academic work in HPE, regardless of previous careers. This includes current post graduate Diploma/Master Health Professions Education students—even if they are proven experts in other fields—graduates, program instructors, authors with initial HPE publications, and active members of international HPE communities seeking deeper research engagement.

ECRs drive innovation and sustainable evidence-based education of the future healthcare workforce. This is particularly important because teaching in HPE is unique; it is not only about imparting knowledge but also about shaping the competencies, and attitudes that directly impact patient care, health outcomes and the wellbeing of society in real-world settings (15). ECRs not only conduct research on education practices in different settings but also develop them based on the new empirical and theoretical knowledge gained. They enrich the discipline with their diverse perspectives and experiences and shape its future (16, 17).

However, developing a research career in HPE is not self-evident or easy, especially given the lack of established and communicated opportunities and career pathways. Although some career development programs for medical education have been established internationally (18), HPE career pathways beyond medicine still lack supportive structures and financing (19). Most importantly, career pathways in HPE are geared primarily toward advanced teaching roles and faculty development responsibilities (20), and less toward a career in educational research (18).

Given this background, our aim was to convene a group of recognized HPE experts to debate and analyze this situation and to explore future directions for ECRs. A key objective was to reach agreement on the pathways, structures and support mechanisms to enhance career development and to provide guidance for advancing this field by articulating a shared vision and key areas for action. It is important to us that the following remarks are only the beginning of a comprehensive discussion that is worth continuing within the HPE community.

## 2 Approach

In November 2024, a Scoping Workshop, funded by the Volkswagen Foundation, took place in Hannover, Germany. This 2.5-day event was organized as part of the “Next Generation of Health Professions Educators” (nextHPE) research project by the Charité—Universitätsmedizin Berlin, Institute of Health and Nursing Science, Germany, in close collaboration with the Zurich University of Applied Sciences, Switzerland.

Scoping Workshops are a format designed to stimulate discussion and exchange of ideas on the current state and key challenges in a specific discipline or research area—in this case, HPE—and to promote the development of new ideas for future research and innovation. For this purpose, we invited senior researchers and scientists, well-recognized for their work in medical, nursing, interprofessional, and allied health education to attend. Nineteen experts with a professional background in education, medicine, midwifery, nursing, occupational therapy, physiotherapy, psychology and public health, participated—some of them had several degrees and represented more than one discipline. They were coming from Australia, Canada, England, Finland, Germany, Ireland, Malta, the Netherlands, Switzerland, the United Arab Emirates, the USA and Wales.

To structure the discussion, we used Appreciative Inquiry (21). This method draws on the creativity and strengths of the people involved, encourages free thinking and improvisation, and promotes collaborative and discursive consensus building. We used a five-step process: (1) definition, (2) discovery, (3) dream, (4) design, and (5) deliver. Following a keynote speech from one of the invitees (ME) and instruction and guidance from a moderator, each phase involved interactive small-group sessions framed by an overarching question to provide a thematic focus, to encourage in-depth exploration and the exchange of ideas (Appendix 1). Collaborative writing sessions (22) were scheduled between phases for systematic documentation. Regular plenaries allowed for cross-group feedback, integration of perspectives, and consensus building.

The diversity of participants facilitated a plethora of multi-dimensional exchange of ideas, allowing for both multidisciplinary, multiprofessional insights and for the inclusion of different international contexts in the deliberations. Due to the composition of the expert group, differences were sensitively considered in the discussions (e.g., conceptual, cultural and linguistic aspects). For example, the word “scholar” was not known to everyone, nor does it have the same meaning everywhere. The participants exchanged views on these and similar points in a targeted manner in order to create a common understanding.

This commitment to heterogeneity is also relevant in view of the diverse audience who will reflect on the outcomes. Our position statement is directed toward researchers, educators, administrators, health and social care providers, politicians and funders with an interest in academia, health and education. They also target editorial board members of HPE journals and members of accrediting bodies. Also, other interested parties in the topic may benefit.

## 3 Shared vision and positions

**Figure 1:**
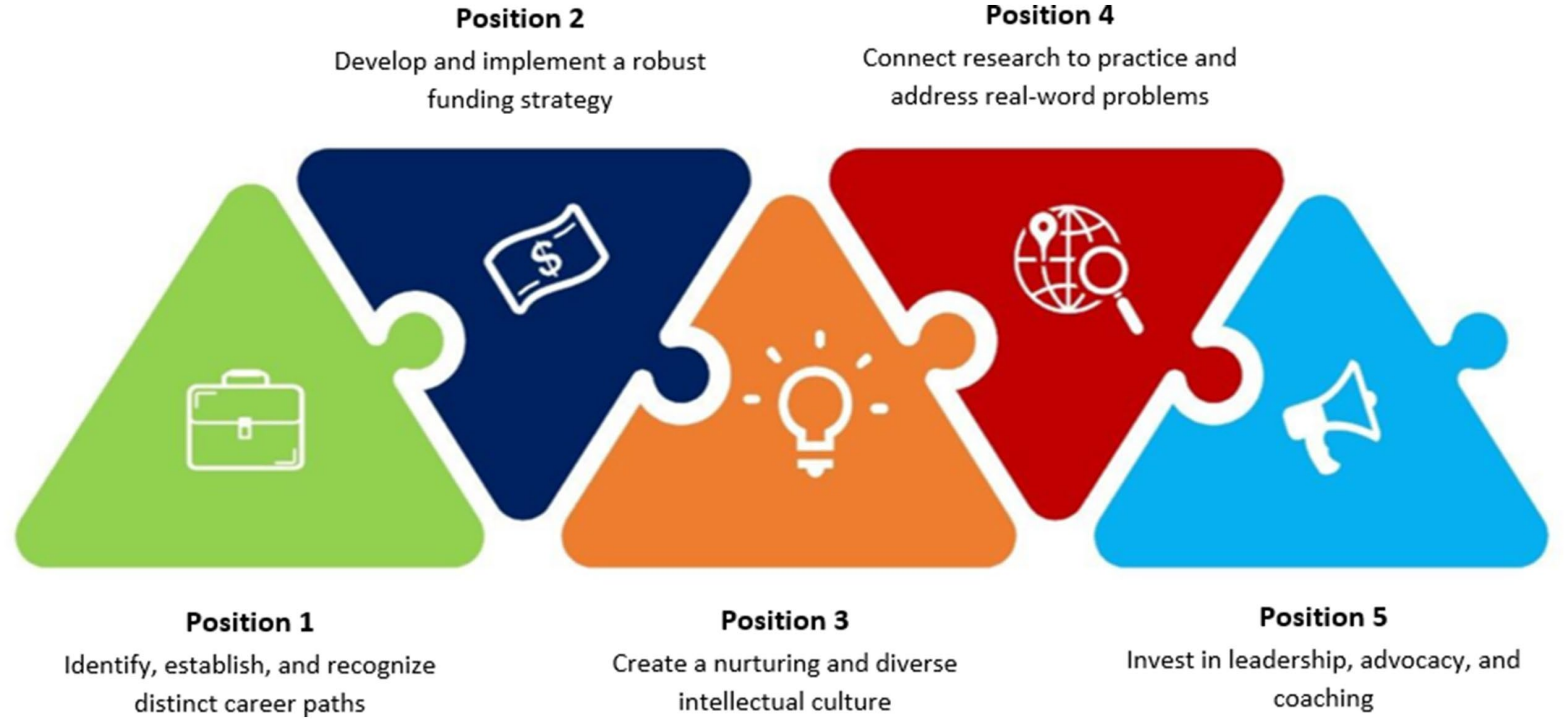
Central positions for the promotion of ECRs in HPE.

Below we present five positions that are the result of our collaborative working process, each with specific implementation steps. The five positions are summarized in Figure 1. The starting point is a jointly developed vision for effective and sustainable career paths in HPER:

*Without a well-educated, competent, flexible and diverse health and social care workforce, health and social care systems will not be able to meet the changing needs of today’s global populations. Good HPE will advance teaching of healthcare professionals, and the new learning will be taken into practice to improve health and social care; educational research will be therefore at its core. In the future we advocate for a* var*iety of visible education research career pathways that will be well-funded, rewarded and extend to the highest levels of organizational leadership. HPER will be intrinsically valued as a way of ensuring optimal health care delivery, in the same way that clinical research is seen to improve practice. Decision-makers will support HPER when it is clear how its results will bring about concrete improvements in the healthcare system and address current challenges in healthcare. Consequently, HPER will be attractive to a diverse range of ECRs, regardless of age, gender or other personal characteristics, disciplinary or professional background, or organizational seniority.*

*Looking to the future, both higher education institutions and health care organizations will even more than now value competent health professions educators and those who invest in evidence-based educational practice. Leadership in these organizations therefore must support and invest in the full pipeline of HPER: from blue-sky to applied knowledge, from knowledge production to knowledge application and integration, and from formal instruction to informal learning in the workplace.*

*In our envisioned future, thriving, well-funded, nationwide HPER and education hubs will support a wide range of research and knowledge integration approaches. They will sustainably enable innovation and cutting-edge basic and applied research; promote (inter-)nationally compatible, interdisciplinary and interprofessional research. With this research, the hubs will provide a strong foundation for ECRs to learn and build a scholarly community and contribute to the translation of knowledge into practice and vice versa,* i.e.*, recognizing the current trends and patterns from practice to gain new insights and create “practical” knowledge/theories. There is countless evidence of educational learning contributing to advances in patient care* (23, 24) *and an ever-growing impetus for practitioners to learn together to advance care; therefore, there will be a strong willingness to prioritize learning, for example interprofessional education* (25).

### 3.1 Position 1: identify, establish, and recognize distinct career paths

It is essential to identify career pathways and to recognize these within a diverse range of roles for all health and social care professionals. A thorough analysis of the labor market may identify the needs and employment opportunities for graduates at an early stage and thus ensure their employability. Currently, there is a lack of clarity regarding career options in HPE, often leading to uncertainty and disillusionment for ECRs. Defining and communicating distinct career pathways in this field will not only serve to enhance the motivation of researchers but also facilitate the integration of education research as a legitimate academic career.

Opportunities for educational research should be made available at an early stage, even during undergraduate/pre-licensure study. Systematic integration throughout the entire training period either as a health professional or as a health professions educator should follow, for example through an HPE certificate, diploma, master’s and doctoral program. Such structuring formalizes entry into the field of educational research. Concurrently, organizations must present appealing career options not only in clinical research and care but also in teaching, learning and educational research, to also compete with other employers. It is particularly important to provide ECRs with transparent career pathways that offer job security. Uncertainty caused by temporary contracts and short-term financing can be mitigated through the provision of targeted support and advocacy for faculty recognition.

It is imperative that educational research in the health and social care professions is afforded the same level of recognition and remuneration as basic or clinical research (*see also position 5*).

*Implementation steps*:

- Introduce teaching and learning principles, theory, and research approaches within all undergraduate/pre-licensure programs.
- Highlight the multiple entry points for HPER career and offer formal pathways such as graduate and postgraduate qualifications, Diploma, and Masters approved by accreditation bodies as well as PhD.
- Aim to offer competitive, stable career paths over the next decade, with seamless transitions from PhD to tenure-track roles.
- Highlight exemplary research careers in HPE—for example, through personal testimonials from faculty members or health organizations—to inspire ECRs by providing mentors, fellowships, opportunities for sabbaticals.
- Tailor training to diverse research roles and recognize excellence in HPER with awards to increase the visibility of this specific field of research and development leading to promotion opportunities.

### 3.2 Position 2: develop and implement a robust funding strategy

Adequate and reliable funding is crucial to support HPER. Current funding models in the context of health often favor biomedical or clinical research, leaving educational research underfunded and undervalued. The distribution of funding for health and educational research has been insufficiently studied (26). This deficiency renders it challenging to make well-informed decisions regarding the allocation of resources. Large grants are highly competitive while many are small in monetary value and so impeding impactful research. A lack of funding for HPER, or even its reduction, can have a considerable adverse impact, such as limited teaching capacities or reduced research activities, that weaken existing structures and prevent necessary innovation.

Investments in HPER are required to facilitate the development of innovative solutions to the complex challenges currently facing the healthcare sector. HPER might have an exponential impact on healthcare practices, as compared to clinical research. Unlike clinical studies which aim to advance diagnostic and management protocols, educational research advances the practices of curriculum development, teaching, learning, instructional design, assessment, and supervision, which contribute to shaping future healthcare practitioners.

Furthermore, it is essential to allocate resources to encourage emerging talent in this field for both immediate benefit and long-term sustainability. Such resources are essential for maintaining the high standards of research, leadership and education.

*Implementation steps*:

- Establish grants for ECRs to motivate them to enter HPER and development.
- Establish a sustainable funding system for HPER (e.g., by allocating a percentage of the tuition fees of medical schools for HPER), which also allows for stable and longer-term financing of research activities.
- Fund multiple tracks, part-time and full-time education in HPER, to attract a diverse group of ECRs.
- Encourage people to investigate research in HPE in the institutional context.
- Ensure practice organizations value the importance of educational researchers who can also maintain caring roles.
- Research the distribution of funding for HPE.

### 3.3 Position 3: create a nurturing and diverse intellectual culture

Support for HPER should be both equitable and sustainable, reflecting the diversity of the health and care context. It is essential to create a health research workforce that includes diverse perspectives and is grounded in solid theoretical and conceptual frameworks.

A supportive ecosystem must be developed, one that values a variety of contributions. Research should focus on understanding the characteristics of successful mentoring in HPE, and investment in faculty development is crucial. Guidance from experienced principal investigators and researchers coming from different theoretical and methodological backgrounds is invaluable, not only for professional growth but also for maintaining the mental wellbeing and sustainable development of ECRs.

Special care should be taken to protect vulnerable groups, including students, postdocs, and those in precarious employment. Although they play a vital role in shaping research outcomes, they often lack the authority and visibility of senior researchers, limiting their ability to fully claim ownership and recognition for their contributions. Ensuring their wellbeing and supporting their contributions will support the long-term success of HPER. ECRs may need to be enrolled in postgraduate programs, either as students or tutors, to feel belonging to the larger HPE community and contribute to support colleagues at various levels of their career progress (27).

*Implementation steps*:

- Protect time and allocate resources to all stages of HPER and scholarship.
- Update policies and procedures to address inequity, and include attention to equity, diversity, and inclusion in appraisals.
- Set up educational research centers in higher education institutions.
- Promote research of effective mentoring strategies, integrate mentorship training and coaching within HPE programs and recognize faculty for mentoring.
- Embrace ECRs within the bigger community of HPE, including senior researchers, editors, leaders in HPE.

### 3.4 Position 4: connect research to practice and address real-world problems

It is necessary to ensure that HPER is relevant, applicable, and impactful. HPER can bridge the gap between research and practice to solve real-world healthcare challenges. To provide significant value, HPER must dynamically respond to these challenges and drive innovation to develop new educational solutions. Key areas of focus include, e.g., educating for digital health integration, crisis and disaster management, addressing workforce shortages and critical societal problems, establishing new patterns for interprofessional collaboration and preparing for emerging healthcare roles (25). To gain the support of decision-makers, HPER must continuously and convincingly demonstrate the concrete benefits of its findings for improving the healthcare system and addressing pressing healthcare challenges.

Collaboration with educational practitioners is crucial to identify the most pressing research questions and ensure that research outputs have tangible, real-world applications, contributing to the impact of education in practice. Opportunities within local, national, and international networks must be clearly defined, enabling health professions educators to bring forward their questions and collaboratively generate new research insights.

Interprofessional and interdisciplinary collaborations are essential to build a robust community of practice and ensuring the scalability of research efforts (28). Strengthening the links between educational institutions, educators, and relevant partners will further enhance the impact and relevance of HPER. Integrating knowledge and theory from diverse disciplines and professions is critical for advancing the field, fostering shared understanding, and promoting innovation.

Collaboration between institutions, disciplines, professions, and nations advance HPER. Researchers who move beyond their home institutions after completing their PhDs gain exposure to different research environments, methodologies, and theoretical perspectives. An (inter-)national experience enables them to bring back new approaches and ideas that can push the boundaries of research, create cutting-edge knowledge, and help advance the HPE field globally. By fostering these connections, the potential for impactful and transformative HPER is greatly enhanced.

*Implementation steps*:

- Describe the impact and extent of HPE on downstream outcomes for people providing and/or receiving health and social care.
- Create a publication strategy that informs practice, policy, and theory equally.
- Facilitate relevance of HPER through the establishment of roles that combine clinical practice and educational research.
- Create a collaborative work environment that lays foundations for researchers’ careers, including mobility possibilities.
- Connect local HPER to external profession specific and interprofessional research networks.
- Connect ECRs with scholars beyond their own professional background, their local institution, and support them to attend and present at local, national and international conferences.

### 3.5 Position 5: invest in leadership, advocacy, and coaching

Leadership and advocacy are crucial to advancing the field of HPER (29). Leaders at all levels should advocate for advancing educational science (as a field) and its applicability to HPE and by this to enhance the visibility, legitimacy and value of this work. The position of HPER within organizations and at all levels of leadership needs to be strengthened. A lack of understanding of the relevance of educational research not only jeopardizes its future development but also the possibility of reaching an equal footing with clinical research. It also deters ECRs from pursuing a career in HPER. Agreements, guidelines and policies at the highest level of universities and health care organizations are required to strategically position HPER alongside clinical research and ensure the necessary institutional support.

In addition to structural anchoring, a bottom-up approach to leadership and coaching is required. This approach facilitates the active involvement of ECRs in HPER decision-making processes, incorporating their perspectives and thereby fostering a culture of shared responsibility. They might be currently leading their institutions in drafting educational policies, chairing committees, leading accreditation of programs, and acting as reference people and experts in HPE. Leaders should identify students interested in educational research, mentor them and offer opportunities to progress their career (30). The promotion of critical thinking and cooperative working methods facilitates the creation of synergies that enhance both individual research skills and collective knowledge sharing. The establishment of a collaborative environment increases motivation and ownership among researchers, while also enhancing the long-term innovative capacity of the entire discipline. Practical support for ECRs in proposal writing, ethics applications, project management, and academic writing would assist in overcoming common early barriers.

The combination of strategic anchoring at the management level and participatory leadership thus provides a solid foundation for the sustainable development of HPER. It is not enough to make only institutional adjustments; a rethinking of the organizational culture is also needed. Currently, it is mostly voluntary providers of educational leadership who support special interest groups and promote collaboration and networking (31, 32).

*Implementation steps*:

- Promote the vital importance of HPER in leadership programs.
- Develop and provide formal training programs for individuals seeking leadership positions in HPER.
- Implement a strengths-based approach to promoting individual and collective competencies for ECRs in HPE.
- Ensure that leaders prioritize research skills when filling positions in HPER.
- Protect the research time of HPER and advocate for additional resources when new responsibilities arise.
- Clinical research programs and grants should include dissemination strategies that specifically include educational activities in practice settings.
- Support ECRs in grant writing, project management and essential research skills like ethics navigation and publication management.

## 4 Discussion and outlook

It can be easy to get lost in the difficulties that befall ECRs who enter the field of HPER—we surfaced multiple issues during our Scoping Workshop, from lack of clarity about professional identities and career pathways and lack of recognition, through to funding limitations, time constraints, and precarious employment resulting in reduced salaries. However, we decided to neither focus on problems nor to initiate an in-depth analysis of the literature on ECRs in HPE. Instead, we started our collaboration with a rather optimistic premise: that a multidisciplinary and multiprofessional group of senior experts from around the world can come together, and, through intensive exchange and critical reflection, they can envision pathways, structures and support mechanisms to enhance career development for ECRs in HPER and reach consensus on promising steps forward.

The result of this process is our position statement, on which we invite discussion from the broader HPE community. It lays out a series of transformative concrete steps. They can enable real change from within institutions, be they universities, health and care organizations, professional societies, etc., and across institutional, professional and disciplinary boundaries, leading to better health and social care. Conferences could be a suitable forum for continuing this conversation and this discourse. The HPER community has a responsibility to help shape the future of ECRs in the field of HPE and to support and safeguard their valued contributions, whether through our work with students, colleagues or wider educational institutions, through teaching, research or leadership on different levels, the actions of everyone are critical. Through the collective commitment of researchers, educators, practitioners, leaders and decision-makers we can ensure that the next generation of researchers can contribute to HPE that is designed to meet the evolving needs of society and to advance the quality of patient care.

## Data Availability

The original contributions presented in the study are included in the article/supplementary material, further inquiries can be directed to the corresponding author.

## Appendix 1

**Table A1:** Guiding questions in each of the working phases.

Working phase	Guiding question
Definition	Which future directions for the promotion of ECRs in HPE do you broadly have in mind when thinking about your own institution?
Discovery	What positive experiences have you or your organization had in supporting junior researchers in HPE, and what approaches have proven effect?
Dream	What would our ideal way of promoting careers in HPE look like?
Design	Which short- and long-term goals are necessary to create support structures which are (i) efficient, (ii) effective and (iii) sustainable?
Deliver	Describe the way to achieve the goals. What specific steps need to be taken?
