# Correction: The support of early-career researchers in health professions education—an expert position statement

**DOI:** 10.3389/fmed.2025.1652208

**Published:** 2025-07-10

**Authors:** Doreen Herinek, Franziska Matthes, Mohamed Al-Eraky, Elizabeth Anderson, Julie Browne, Maria Cassar, Ingrid Darmann-Finck, Götz Fabry, Marion Huber, Fiona Kent, Mirjam Körner, Sylvia Langlois, Kristina Mikkonen, Elise Paradis, Lisa Quinn, Ara Tekian, Daniëlle Verstegen, Robyn Woodward-Kron, Michael Ewers

**Affiliations:** ^1^Institute of Health and Nursing Science, Charité – Universitätsmedizin, Berlin corporate member of Freie Universität Berlin and Humboldt-Universität zu Berlin, Berlin, Germany; ^2^Institute of Health Workforce Development, Gulf Medical University (GMU), Dubai, United Arab Emirates; ^3^Leicester Medical School, College of Life Sciences, The University of Leicester, Leicester, United Kingdom; ^4^School of Medicine, Cardiff University, Wales, United Kingdom; ^5^Faculty of Health Sciences, University of Malta, Msida, Malta; ^6^Institute for Public Health and Nursing Research, University of Bremen, Bremen, Germany; ^7^Institute for Medical Psychology and Medical Sociology, University of Freiburg, Freiburg, Germany; ^8^Center for Interprofessional Learning and Practice IPLP, School of Health Sciences, Institute of Public Health IPH, Zurich University of Applied Sciences (ZHAW), Winterthur, Switzerland; ^9^Royal College of Surgeons in Ireland, University of Medicine and Health Sciences, Dublin, Ireland; ^10^Institute for Collaborative Practice and Leadership in Healthcare, University of Applied Sciences Bern, Bern, Switzerland; ^11^Centre for Advancing Collaborative Healthcare and Education, University Health Network and University of Toronto, Toronto, ON, Canada; ^12^Research Unit of Health Sciences and Technology, University of Oulu, Oulu, Finland; ^13^NONE, San Francisco, CA, United States; ^14^College of Medicine, University of Illinois, Chicago, IL, United States; ^15^School of Health Professions Education, Faculty of Health, Medicine, and Life Sciences, Maastricht University, Maastricht, Netherlands; ^16^Department of Medical Education, Melbourne Medical School, University of Melbourne, Melbourne, VIC, Australia

**Keywords:** health professions education, early-career researchers, position statement, medical education, career pathways

Affiliation “Research Unit of Health Sciences and Technology, University of Oulu, Oulu, Finland” was erroneously given as “Centre for Advancing Collaborative Healthcare and Education, University of Oulu, Oulu, Finland”.

The original version of this article has been updated.

